# Preprocessing of natural language process variables using a data-driven method improves the association with suicide risk in a large veterans affairs population

**DOI:** 10.1016/j.compbiomed.2025.109939

**Published:** 2025-03-05

**Authors:** Siting Li, Maxwell Levis, Monica DiMambro, Weiyi Wu, Joshua Levy, Brian Shiner, Jiang Gui

**Affiliations:** a Department of Biomedical Data Science, Dartmouth College, Hanover, NH, USA; b Department of Veterans Affairs Medical Center, White River Junction, VT, USA; c Pathology and Computational Biomedicine, Cedars Sinai Medical Center, Los Angeles, CA, USA; d Department of Psychiatry, Dartmouth College, Hanover, NH, USA

**Keywords:** Electronic health record, Suicide risk, Natural language processing, Categorization, Logistic regression, Effect size

## Abstract

**Objective:**

Suicide risk assessment has historically relied heavily on clinical evaluations and patient self-reports. Natural language processing (NLP) of electronic health records (EHRs) provides an alternative approach for extracting risk predictors from clinical notes. Modeling NLP variables, however, is challenging because of zero inflation and skewed distributions. Therefore, we evaluated whether an adaptive-mixture-categorization (AMC) method could optimize the suicide risk predictive capacity of NLP data extracted from Veterans Affairs (VA) EHR notes.

**Methods:**

NLP variables for 25,342 patients were analyzed using the SÉANCE python package. The AMC method was employed to categorize NLP measures into distinct groups to maximize the between-category variance. Associations between suicide outcomes and AMC-categorized NLP variables were compared to those between the original and quantile-categorized NLP variables.

**Results:**

AMC-categorized variables showed stronger associations with suicide risk than other approaches did in the full cohort analysis and sensitivity analyses by subsampling bootstrapping. Additionally, over 90 % of the NLP variables were significantly associated with suicide risk in univariate analyses, indicating the relevance of clinical notes in suicide prevention.

**Conclusion:**

AMC-based categorization substantially enhanced the suicide predictive capacity of NLP variables extracted from clinical text. Transforming skewed NLP data with the AMC method holds promise for improving risk prediction models.

## 1. Introduction

Suicide is a major public health concern, with over 47,000 deaths caused by suicide occurring in the United States in 2019 alone [1,2]. Given the complexity of the factors contributing to suicide risk, developing accurate risk prediction models remains an ongoing challenge [3]. Traditional approaches to suicide risk screening rely heavily on clinicians’ assessments and patient self-reports, which can be limited by issues such as disclosure reluctance and interpretation biases [4–7]. To address these limitations, recent studies have explored the use of natural language processing (NLP) methods to extract additional information from electronic health record (EHR)-free text to aid suicide risk analysis [7,8].

NLP includes a range of computational methods designed to analyze unstructured text data automatically [9]. These methods include tasks such as processing, feature extraction, and sentiment classification [9]. By applying NLP algorithms to EHR notes and other clinical text documents, variables relevant to suicide risk assessment can be systematically extracted and analyzed. For example, NLP methods can identify terms and phrases in clinician notes that reveal heightened states of anxiety, feelings of burdensomeness, or recent stressors that signal elevated suicide risk [7].

Early investigations demonstrated the potential for the application of NLP algorithms to EHR text data to increase suicide risk modeling accuracy beyond what structured EHR fields allow [10]. NLP analysis of EHR notes may enable more dynamic tracking of fluctuations in suicide risk over time [11] and the identification of personalized risk factors such as therapeutic alliance quality [12] or a lack of goal-directed reasoning [10] that are not otherwise feasibly quantified.

By providing an additional channel for accessing clinically meaningful risk variables, NLP methodologies enhance suicide screening, prevention, and treatment efforts [10,13]. However, many NLP variables have a high proportion of zeros (i.e., most documents lack the majority of the concepts that could potentially be detected with NLP), leading to zero-inflated distributions, whereby the number of zeros is greater than we would expect in typical distributions of counts used in statistics (e.g., Poisson distribution or negative binomial distribution). Simply excluding zeros or ignoring zero inflation can result in biased estimates. On the other hand, keeping zeros in the regression model can increase the variance of the error terms, violates the homoscedasticity assumption of linear regression. Furthermore, the relationship between the predictor and the response variable may not be linear when there are many zeros. This nonlinearity can make the linear model a poor fit for the data.

Previous studies [7,8,14–19] have failed to directly address zero inflation in NLP variables. Instead, NLP variables are combined using either unsupervised [14] or supervised methods [15–17]. In this way, the combined predictor overcomes the zero-inflation problem. However, the individual effect of each NLP variable is overlooked, which reduces the interpretability of the results. Furthermore, proper preprocessing of the NLP variables can improve their association strength and lead to more powerful combined predictors.

The adaptive-mixture-categorization (AMC) method [20] provides a flexible framework for handling zero-inflated data in regression analysis. AMC can optimally categorize NLP variables into groups that maximize between-group variance using an F statistic without needing to specify percentile cutoff points *a priori*. By separating the large cluster of zeros and low values from higher values, AMC generates categories that reflect the underlying mixture distribution with monotonic and nonlinear effects [20]. Alternative methods, such as quantile-based or discretization techniques, have been used to address zero inflation but often fail to differentiate risk in the upper distribution tails. Our AMC approach provides a novel solution that retains essential variance while enhancing interpretability. This approach addresses a longstanding challenge and offers a unique contribution to the field by improving risk prediction accuracy.

This study explores the potential utility of AMC-transformed NLP predictors from Veterans Affairs (VA) EHR notes in suicide risk assessment. Optimizing the integration of structured and unstructured EHR elements can profoundly strengthen suicide screening and prevention initiatives.

## 2. Methods and materials

**Fig. 1.**
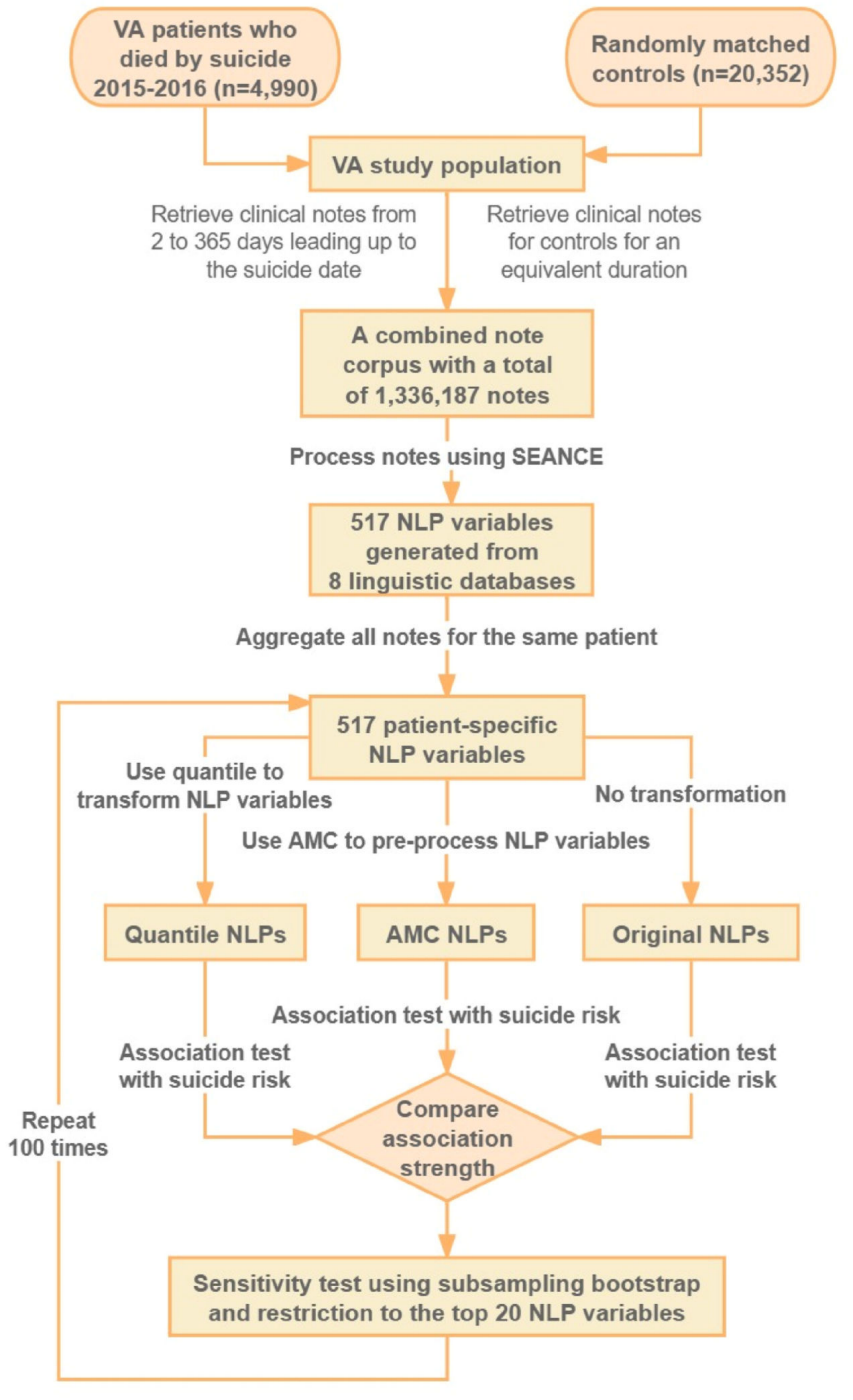
Study outline.

Fig. 1 presents the methods employed to identify the associations between clinical notes processed by NLP and death by suicide. Section 2.1 introduces the VA suicide cohort and how we processed clinical notes using a Python-based NLP package. Sections 2.2–2.3 describe the AMC method and its application to the VA suicide cohort. Section 2.4 outlines the statistical analysis used to associate AMC-categorized NLP variables with suicide outcomes and the comparison of the association strength with the original NLP variables and quantile-categorized NLP variables. Section 2.5 demonstrates the sensitivity analysis used to test whether the conclusion would change in scenarios involving small to medium sample sizes.

### 2.1. Study population and predictor extraction

As described by Levis et al. [7], patients were selected by integrating EHR data from the VA’s Corporate Data Warehouse (CDW) with mortality information from the VA/Department of Defense (VA/DoD) Mortality Data Repository [21]. We first identified individuals who died by suicide and had one or more medical visits at the VA in 2015 or 2016, resulting in a total of 4990 cases. The control group consisted of VA patients who did not die by suicide during this period. Using a random matching strategy, each case was matched with four controls, resulting in a total of 20,352 controls. Cases were assessed for up to one year preceding the date of suicide, whereas controls were assessed for an equivalent duration aligned with the date of death of their respective case. All medical visit notes within this timeframe were extracted from the CDW without imposing any restrictions on size or length. To reduce potential endogeneity, notes from within two days before the suicide event were excluded, as death dates can be inaccurate by a few days, and this period may include post suicide communications with or from families. Controls with more than three times the mean number of notes were excluded to prevent bias toward patients with higher visit frequencies. In total, we included 1,336,187 notes in our analysis. Demographic information can be found in Table 1.

We utilized the sentiment analysis and cognition engine (SÉANCE) [22] to process notes. SÉANCE is a supervised semantic analysis package based on Python that employs multiple linguistic databases [23–29]. These resources include rule-based systems and expert-derived lexicons [30]. We generated patient-specific NLP predictors by aggregating all the notes related to the same patient and summed all frequencies of SÉANCE-derived NLP terms to reduce zero inflation and remove time dependence in these variables.

### 2.2. AMC method

We employed the AMC method to preprocess the NLP variables and address the issue of zero inflation. As described by Li et al. [20], the AMC method converts continuous variables to categorical variables using data-driven thresholds by identifying a set of thresholds that maximize the ratio of between-category variance to within-category variance, thus retaining the maximum variance after the transformation. This method aims to identify an optimal categorization rule *f*(*n*, *k*) to categorize the variable of length *n* into *k* categories (*k* ≥ 2). This process involves searching for a set of thresholds that minimizes the loss function *L*(*f*(*n*, *k*)), which is defined as the ratio of within-category variation to between-category variation [20]. The loss function is the reciprocal of the F statistic [31,32] from an analysis of variance (ANOVA) test.

The AMC method uses a linear search strategy to find the optimal number of categories *k*. This approach reduces the computational complexity compared with that of an exhaustive search, as outlined in a previous study [20]. The search begins with *k* = 2 to identify the best threshold for splitting the data into two categories. Next, the method proceeds to *k* = 3, searching for another threshold to divide the data into three categories while keeping the previous threshold fixed. Each subsequent threshold for the *k*th category is determined by fixing the thresholds from the previous steps. The optimal *k* value is determined by minimizing the p value obtained from the F test. Although the loss function monotonically decreases with *k*, the degree of freedom in the F test also increases with *k* so that the p value does not monotonically decrease with *k*.

In this study, we applied the AMC method to categorize all the NLP variables. Both thresholds and the number of categories were determined by p values from F tests. Given the right skewness of the NLP variables, the number of samples in categories on the right tail tended to be sparse. Therefore, we combined all the sparse categories (n < 20) with the nearest neighboring categories on the left until all the categories had more than 20 samples.

### 2.3. Computational burden and an efficient alternative

There is a considerable computational burden in searching for up to 10 thresholds for large datasets using the linear search strategy of the AMC method. In our case, there are n = 25,342 patients and p = 517 SÉANCE-generated NLP variables. The AMC method may need to calculate the loss function n × p × k = 1.3 × 10^8^ times, which would require more than 500 h to run in the VA Informatics and Computing Infrastructure (VINCI) server environment. To increase the efficiency of the AMC method, we limited the search to the top 1000 largest data gaps, defined as the differences between adjacent values in the ordered data for each threshold. The rationale is that breaking a larger gap in the data can reduce the loss function more than breaking a smaller gap when other data points remain the same. We found that this alternative search strategy yields results close to those from the full-scale linear search. This strategy can reduce the computational burden by more than twentyfold, enabling smooth execution on the VINCI platform.

### 2.4. Comparing AMC-categorized NLP variables with original and quantile-categorized NLP variables for association strength with suicide risk

We first treated AMC-categorized NLP variables as categorical variables and compared them with quantile-categorized NLP variables. We conducted the chi-square test to identify p values between AMC-categorized NLP variables and suicide status. The same process was repeated for quantile-categorized NLP variables, resulting in a new set of p values. We also calculated phi effect sizes [33] for both scenarios. Next, we treated AMC-processed NLP variables as ordinal variables and assumed that the risk differences between adjacent categories were equal. For each variable, we conducted a test for a linear trend to evaluate its association with suicide risk. Given the dichotomous outcome, we used logistic regression for linear trend tests. We obtained three sets of p values for ordinal AMC-categorized NLP variables, ordinal quantile-categorized NLP variables, and original NLP variables. We then calculated Cohen’s effect sizes [34] for the above three scenarios.

Since the NLP variables are identified using multiple linguistic libraries, we also compared the five sets of p values above within four major text categories: Harvard IV-4 (238 NLP variables), Lasswell (126 NLP variables), Genva affect label coder (76 NLP variables) and SÉANCE component scores (20 NLP variables). Additionally, we compared mean effect sizes across all individual NLP variables and within text categories.

### 2.5. Sensitivity analysis using bootstrap sampling

We performed a sensitivity analysis using bootstrap samples [35] without replacement. We undersampled [36] the cases to 500 and 1000, with 2000 and 4000 controls, respectively. We repeated the analysis 100 times using the top 20 NLP variables identified by the AMC categorization. We compared the average association strength between the AMC-categorized variables and the quantile-categorized variables using p values and phi effect sizes.

## 3. Results

In this study, we included 4990 suicide cases and 20,352 controls. The mean age of the participants was approximately 60 years, and most participants were male (~90 %). As shown in Table 1, patients were more likely than controls were to have mental health issues such as depression and anxiety. In addition, higher percentages of homelessness, unmarried status, and nondisability were observed among the cases than among the controls. Differences between cases and controls were small, as indicated by standardized mean difference (SMD) values according to the criteria described by Andrade [37]: SMD values between 0.2 and 0.5 were defined as small values, those between 0.5 and 0.8 as medium values, and those greater than 0.8 as large values.

We did not use structured data, including demographic and diagnostic characteristics, to develop the NLP model. The observed differences in the study population reflect the random matching process. We included 517 SÉANCE-coded NLP variables from various text categories. Table 2 outlines the distributions of SÉANCE text categories for the NLP variables.

### 3.1. Associations between AMC-categorized NLP variables and suicide risk

We applied the chi-square test to identify associations between AMC-categorized NLP variables and suicide risk. Table 3 lists the top 20 NLP variables ranked by chi-square p values. Among these, 12 are positive, and 8 are negative. All 8 negative-form NLP variables have corresponding counterparts among the 12 positive-form NLP variables, which is expected, as the positive and negative forms of the same NLP term are highly correlated.

**Fig. 2.**
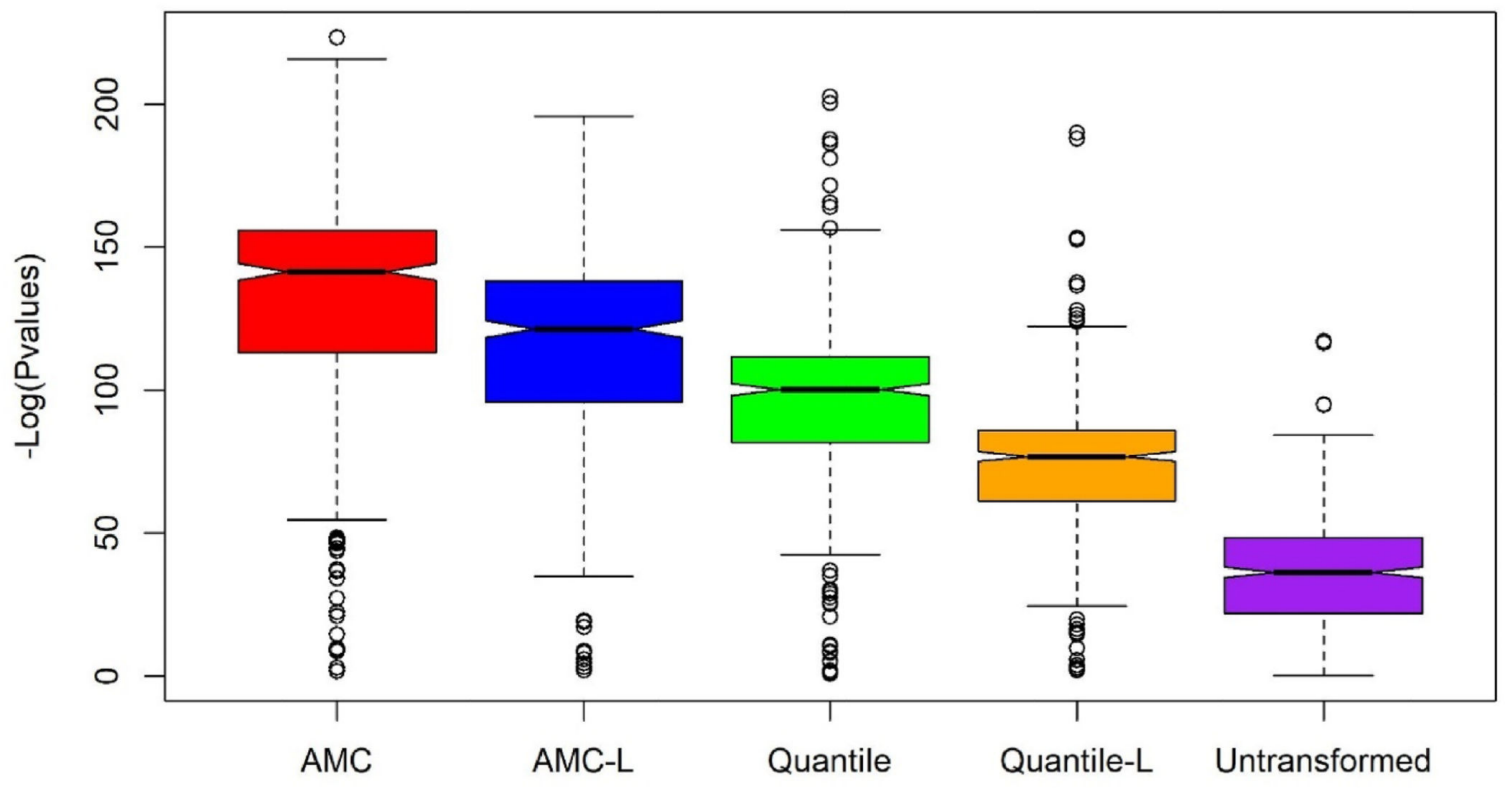
Boxplot of the -log10 p-values for all 517 NLP variables across five methods^a^. ^a^ “AMC” and “Quantile” represent NLP variables were transformed using AMC and quantile categorizations, respectively, and treated as categorical variables, with their -log10 p-values calculated by Chi-squared tests. “AMC-L” and “Quantile-L″ represent variables transformed using AMC and quantile categorizations and treated as continuous variables, with -log10 p-values calculated by linear trend tests. “Untransformed” refers to the original NLP variables, with -log10 p-values calculated by linear trend tests.

**Fig. 3.**
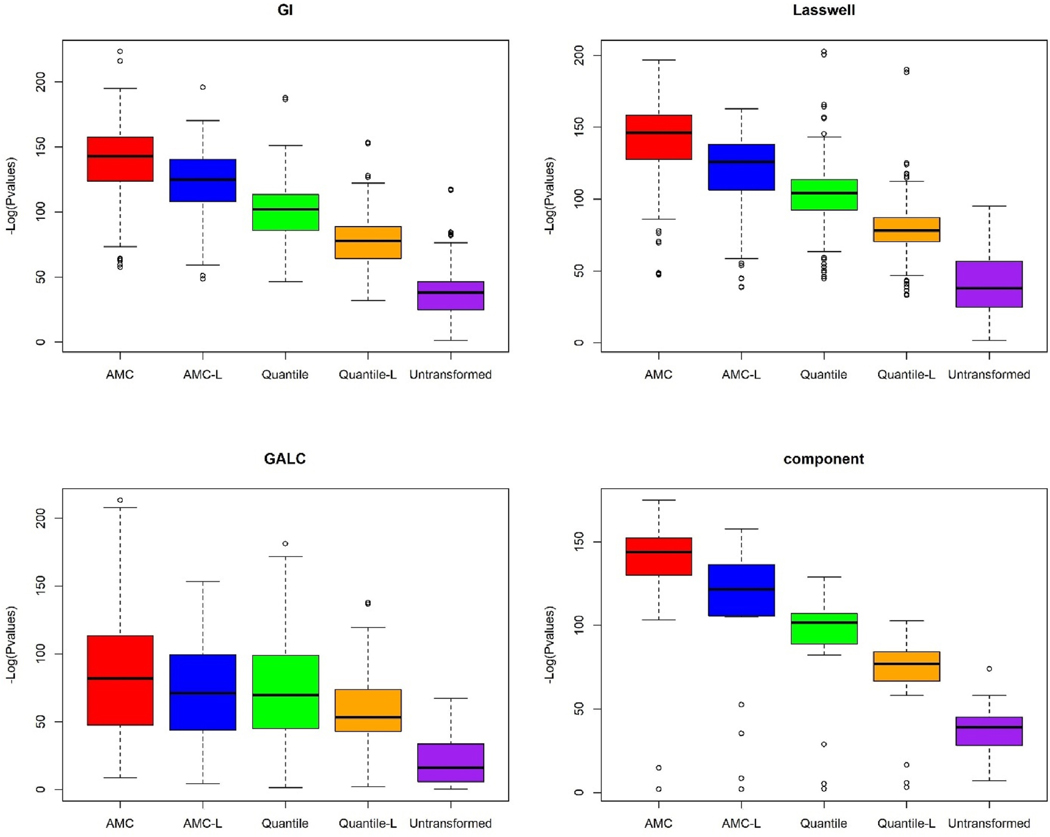
Boxplot of p-value magnitudes from five methods within the four largest NLP text categories^a^. ^a^ GI represents the “General Inquirer (Harvard IV-4)” database with 238 NLP variables. Lasswell database comprises 126 NLP variables. GALC represents the “Geneva affect label coder” database with 76 NLP variables. Component represents the “SÉANCE component scores” database with 20 NLP variables.

We compared the significance levels of the AMC-categorized NLP variables with those of the original NLP variables and quantile-categorized NLP variables. Given that most of the NLP variables listed in Table 3 have a monotonic risk pattern across categories, we treated both categorized variables as continuous measures and used logistic regression to assess their associations with suicide risk. Two new sets of p values for monotonic effects were named AMC-L and Quantile-L, as they test the linear trend for AMC and quantile-categorized variables. In Fig. 2, AMC and AMC-L present the highest overall significance levels, with mean -log10-transformed p values of 130.3 and 112.8, respectively. The next closest results were from Quantile and Quantile-L, with means of 95.5 and 73.5, respectively. The untransformed NLP variables had the lowest overall significance, with a mean -log10 transformed p value of 35.9. We also found the same pattern in terms of significance ranking (Fig. 3) when we compared p values across the five preprocessing methods within the four text subcategories. P values from the Geneva affect label coder (GALC) were less significant than those from the other three categories.

**Fig. 4.**
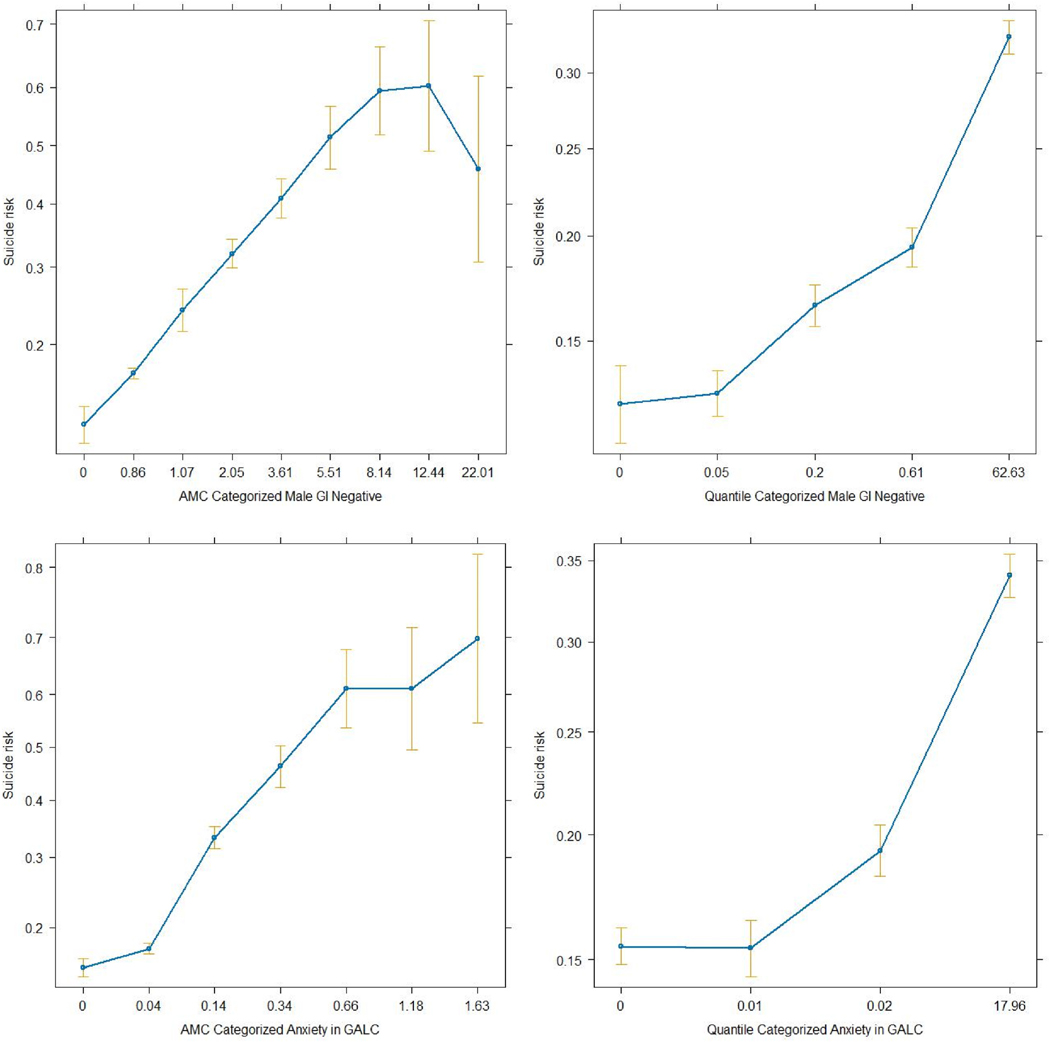
Comparison between AMC-categorized NLP variables, quantile-categorized NLP variables, and suicide risk. Each subplot represents the association between suicide risk and one of the following NLP variables: AMC-categorized and quantile-categorized variables for the negative term “Male” in the GI dictionary, and AMC-categorized and quantile-categorized variables for the positive term “Anxiety” in the GALC dictionary.

We compared the mean effect sizes across the five methods, and the results are presented in Table 2. Similar to the p values, AMC categorization outperformed quantile categorization by 15 % overall and across eight linguistic libraries. This outcome holds true for both categorical and continuous effect sizes. AMC almost doubled the average Cohen’s D effect size of the original NLP variables. To further understand why AMC categorization results in stronger associations than quantile categorization does, we compared the effect plots between AMC and quantile categorization for two of the top NLP variables in Table 3. Fig. 4 shows that AMC effectively captures the linearly increasing risk in the right-tail region. In contrast, quantile categorization tends to consolidate all large values into Q4, disregarding the significant high-risk variances (ranging from 0.3 to 0.7) among them. This finding suggests that AMC is more effective in dissecting granular risk categories in the tail region, thereby enhancing associations in both the linear trend model and the chi-square test.

Previous studies [7,10,14,15] ranging from 2021 to 2024 have emphasized the potential of NLP to improve suicide risk assessment, yet concerns remain over data handling complexities and predictive reliability. By using AMC categorization, we have made substantial progress in overcoming these barriers. Recent advancements in NLP modeling [7, 10,14–17] have not achieved the same level of risk stratification observed with our approach, highlighting the novelty and importance of our method.

### 3.2. Sensitivity analysis with undersampling

Since many suicide studies have fewer than 10,000 participants [38], we evaluated the performance of AMC-categorized variables in a smaller sample size setting (Table 4). We sampled 1000 suicide cases without replacement from the 4990 cases in our data and randomly matched controls at a 1:4 ratio to create an undersampled cohort of 5000 participants. We applied AMC and quantile categorizations to the 20 NLP variables presented in Table 2 and applied the chi-square test to assess their associations with suicide risk. We conducted chi-square tests for both AMC-categorized NLP variables and quantile-categorized NLP variables and calculated how often AMC-categorized NLP variables yielded a more significant p value across 100 sampling iterations. We estimated the average phi effect size and standard deviations for both AMC and quantile categorizations across these 100 iterations. We also evaluated the consistency of our findings by changing the sample size to 500 cases and 2000 controls. As noted in Table 4, AMC categorization outperformed quantile categorization over 90 % of the time in both undersampled settings. Additionally, the average effect size of AMC categorization was approximately 15 % greater than that of quantile categorization, which is consistent with the results from the full cohorts.

## 4. Discussion and conclusion

This study demonstrates the benefit of using AMC to preprocess NLP variables extracted from clinical notes for suicide risk modeling. Our results showed that AMC-categorized NLP predictors had significantly stronger associations with suicide outcomes than did original continuous NLP variables and quantile-categorized NLP variables. The enhanced association of AMC-processed variables held true in both the full cohort analysis and sensitivity analyses using undersampled data.

The interpretability of AMC categories in clinical settings is critical for practical adoption. While the AMC method enhances predictive power by optimizing categorization, it introduces complexity. In clinical practice, quantile-based categorizations are more familiar to healthcare providers. However, AMC categories, though less intuitive, can be simplified by presenting them as ordinal risk levels, allowing clinicians to interpret risk more effectively. Furthermore, transforming AMC categories into continuous variables preserves the granularity needed for nuanced risk assessment without overcomplicating clinical decision-making.

AMC provides an optimized, data-driven approach to categorizing skewed and zero-inflated NLP data in a manner that maximizes between-category variance while retaining important predictive information such that simply excluding abundant zeros or keeping variables continuous may fail to be achieved. As shown in Fig. 4, the AMC method may improve the ability to differentiate language patterns associated with varying levels of suicide risk by categorizing NLP variables into categories with flexible sizes. This study contributes to theoretical frameworks on suicide risk by providing a data-driven approach that enhances the strength of the associations between NLP variables and suicide risk. Our findings support theories suggesting that nuanced text analysis can uncover latent risk factors more effectively. Practically, the AMC method offers a pathway to more accurate suicide risk prediction models that could improve screening and prevention strategies in clinical settings.

On the other hand, data-driven thresholds of AMC categorization are more difficult to interpret than are the original NLP variables or quantile-categorized NLP variables. This can be partially alleviated by treating the categories as a continuous measure. We have demonstrated that there is a strong linear association between numerical AMC categories and suicide risk. Indeed, linear trend p values are almost as significant as the chi-square p values in this cohort. Another advantage of treating categories as continuous variables is reducing the number of parameters. For example, quantile categorization requires three parameters to dummy code the categories in a regression model, and AMC categorization transforms most NLP variables into five or more categories. Using linear trend p values can greatly improve the interpretability of the suicide risk model.

Our cohort included a large sample of VA patients, covering all suicide deaths over a two-year period. Matching each case to multiple controls provided a strong comparison group. We extracted NLP predictors from a broad repository of EHR notes without filtering by note type, offering a rich text corpus for analysis. On the other hand, this study has a few limitations. First, the findings may not be fully generalizable to populations other than Veterans receiving care in the VA health system. Nevertheless, this approach holds promise for broader clinical populations, including Veterans receiving care in non-VA healthcare systems. Differences in healthcare access, patient demographics, and EHR structures could influence the performance of AMC-categorized variables. Future studies should validate our findings across diverse clinical settings to ensure the robustness and adaptability of the method. Expanding the analysis to different healthcare systems will be essential to confirm the generalizability and practical utility of AMC categorization. Second, AMC categorization has a significant computational cost. For example, an exhaustive linear search would require over 20 days to complete as a single job using VINCI, which does not have graphics processing unit (GPU) or parallel computing capacity. Even after the search strategy was optimized, the total run time for the 517 NLP variables was 25.37 h. This limitation will likely be addressed when the VA implements a newer cloud computing system. Although we limited the search to a maximum of 10 categories, a large proportion of the AMC-categorized NLP variables reached the full 10 categories determined by the F test, which suggests a need to increase searches to include a greater number of categories. However, samples are unevenly distributed across these categories. The large value categories always contain far fewer samples than the small value categories do, as outliers tend to have a larger impact on within-group variance, leading the AMC method to group the few outliers into one category. In this study, we collapsed these small categories together to ensure that each category had at least 20 samples, making the final categorization more robust. Third, the implementation of the AMC method in large-scale clinical environments presents computational challenges. We used a high-performance server setup. However, even with this setup, AMC categorization required extensive computational time. For practical deployment, potential strategies include using parallel computing to distribute computing resources and expedite the categorization process. Alternatively, we could be more selective on categorization and only apply AMC to variables with the highest impact on predictive performance to reduce processing needs.

In future work, we plan to refine the AMC categorization method with an alternative strategy for determining the optimal number of categories. Although the F test guards against overfitting by penalizing the number of categories, it does not fully eliminate the ultrasmall categories driven by outliers. We will explore the predictive performance of these NLP variables preprocessed by AMC categorization. We expect those variables to result in greater improvement in prediction performance compared with original or quantile-categorized NLP variables, as AMC categorization can reveal the latent association with suicide risk.

In conclusion, preprocessing NLP variables by applying AMC categorization can improve suicide risk associations using information extracted from clinical notes. Our results suggest that data-driven categorization approaches such as AMC can address longstanding challenges in zero-inflated and skewed distributions, providing more reliable and interpretable risk assessments. These findings pave the way for advancements in suicide prevention efforts, emphasizing the need for continuous innovation in leveraging unstructured EHR data. Identifying and integrating optimally informative predictors is imperative to improve risk screening, monitoring, and prevention in healthcare systems.

## Figures and Tables

**Table 1:** Descriptive characteristics of the study population. Cases were VA patients who died by suicide in 2015 or 2016 and controls were VA patients who did not die during those years.

	Case	Control	Standardized mean difference (SMD)
	(N = 4990)	(N = 20352)	

**Gender**			0.237
Female	230 (4.6 %)	2220 (10.9 %)	
**Age**			0.008
Mean (SD)	59.9 (18.8)	60.0 (16.4)	
Median [Min, Max]	63.0 [18, 100]	64.0 [18, 116]	
Missing	14 (0.3 %)	40 (0.2 %)	
**Marital Status**			0.263
Married	2042 (40.9 %)		10976 (53.9 %)
Nonmarried	2948 (59.1 %)		9365 (46.0 %)
Missing	0 (0 %)		11 (0.1 %)
**Race**			0.329
Am_Ind or Asian_Pac	97 (1.9 %)		584 (2.9 %)
Black	279 (5.6 %)		2993 (14.7 %)
Hispanic	210 (4.2 %)		1119 (5.5 %)
Unknown	492 (9.9 %)		1562 (7.7 %)
White	3912 (78.4 %)		14094 (69.3 %)
**Percent Disability**			0.345
Not Disabled	2760 (55.3 %)		8130 (39.9 %)
0–60 % Disabled	1134 (22.7 %)		4966 (24.4 %)
70–100 % Disabled	1096 (22.0 %)		7245 (35.6 %)
Missing	0 (0 %)		11 (0.1 %)
**Eligibility**			0.371
Not Service Connected	2477 (49.6 %)		6727 (33.1 %)
Service Connected <50 %	932 (18.7 %)		3864 (19.0 %)
Service Connected 50–100 %	1433 (28.7 %)		8770 (43.1 %)
Other	0 (0 %)		11 (0.1 %)
Missing			
**Deployment**			
Vietnam	1727 (34.6 %)	8306 (40.8 %)	0.146
Afghanistan or Iraq	1624 (32.5 %)	6705 (32.9 %)	0.022
**Mental Health Diagnosis/Risk Flag/Homelessness**			
Anxiety	1875 (37.6 %)	5001 (24.6 %)	0.287
Bipolar	838 (16.8 %)	1416 (7.0 %)	0.31
Combat	1212 (24.3 %)	3803 (18.7 %)	0.136
Depression	2731 (54.7 %)	7263 (35.7 %)	0.396
Homeless_prior24m	313 (6.3 %)	688 (3.4 %)	0.135
PTSD	1429 (28.6 %)	4389 (21.6 %)	0.166
Personality	530 (10.6 %)	921 (4.5 %)	0.234
Substance	1701 (34.1 %)	3842 (18.9 %)	0.354
Trauma	2003 (40.1 %)	6238 (30.7 %)	0.202

**Table 2:** The average effect size within each text category in SÉANCE. Effect sizes, including the phi coefficient and Cohen’s d, were calculated for of all 517 NLP variables associated with the suicide risk.

Categories^a^	Number of NLP variables	AMC categorization mean phi	Quantile categorization mean phi	AMC categorization mean Cohen’s d	Quantile categorization mean Cohen’s d	Original/Uncategorized mean Cohen’s d

Lasswell	126	0.161	0.138	0.34	0.297	0.198
GI^b^	238	0.16	0.135	0.342	0.292	0.196
GALC^c^	76	0.121	0.11	0.255	0.234	0.134
Component	20	0.152	0.125	0.29	0.279	0.155
scores						
EmoLex	20	0.167	0.138	0.347	0.295	0.178
VADER^d^	4	0.132	0.114	0.289	0.258	0.125
Hu-Liu	10	0.162	0.132	0.345	0.286	0.188
Special words	23	0.164	0.126	0.291	0.266	0.145
Total	517	0.154	0.131	0.325	0.283	0.182

a Name of linguistic databases/dictionaries.

b GI represents the “General Inquirer (Harvard IV-4)” database.

c GALC represents the “Genva affect label coder” database.

d VADER represents the “Valence Aware Dictionary and sEntiment Reasoner” database.

**Table 3:** A summary of the top 20 AMC-categorized NLP variables for the study population, categorized by suicide outcome. NLP variables in positive forms are listed on the left side and negative forms are listed on the right. Within each side, the first column outlines NLP variable names, and the upper thresholds identified by AMC. The second and third columns outline counts and percentages. Significance tests comparing AMC-categorized variables with suicide risk were performed using the Chi-squared test, with the -log_10_ p-values reported in the fourth column.

NLP variables in positive form				NLP variables in negative form			
AMC threshold	Case count (percentages)	Control count (percentages)	−log_10_p	AMC threshold	Case count (percentages)	Control count (percentages)	−log_10_p

Male (GI)^a^			215.96	Male (GI)			223.37
0.00	264 (5.3)	1847 (9.1)		0.00	266 (5.3)	1858 (9.1)	
1.03	3435 (68.8)	16396 (80.6)		0.86	3222 (64.6)	15738 (77.3)	
2.01	607 (12.2)	1323 (6.5)		1.07	226 (4.5)	712 (3.5)	
3.55	355 (7.1)	507 (2.5)		2.05	593 (11.9)	1258 (6.2)	
5.38	161 (3.2)	155 (0.8)		3.61	353 (7.1)	510 (2.5)	
8.07	105 (2.1)	74 (0.4)		5.51	165 (3.3)	156 (0.8)	
12.34	46 (0.9)	30 (0.1)		8.14	101 (2.0)	69 (0.3)	
21.66	17 (0.3)	20 (0.1)		12.44	47 (0.9)	31 (0.2)	
				22.01	17 (0.3)	20 (0.1)	

Anxiety (GALC)			213.07	Anxiety (GALC)			207.83
0.00	962 (19.3)	5241 (25.8)		0.00	973 (19.5)	5268 (25.9)	
0.04	2778 (55.7)	13099 (64.4)		0.04	2852 (57.2)	13264 (65.2)	
0.14	788 (15.8)	1579 (7.8)		0.15	720 (14.4)	1413 (6.9)	
0.34	278 (5.6)	322 (1.6)		0.36	263 (5.3)	299 (1.5)	
0.66	110 (2.2)	70 (0.3)		0.68	110 (2.2)	67 (0.3)	
1.18	44 (0.9)	28 (0.1)		1.26	72 (1.4)	41 (0.2)	
1.63	30 (0.6)	13 (0.1)					

Affect loss (Lasswell)			196.62	Emotion (GI)			193.86
0.00	2100 (42.1)	12487 (61.4)		0.00	269 (5.4)	1453 (7.1)	
0.01	1792 (35.9)	6050 (29.7)		0.48	3319 (66.5)	16297 (80.1)	
0.03	625 (12.5)	1176 (5.8)		1.15	749 (15.0)	1848 (9.1)	
0.08	289 (5.8)	397 (2.0)		2.59	405 (8.1)	548 (2.7)	
0.11	64 (1.3)	85 (0.4)		4.99	155 (3.1)	143 (0.7)	
0.17	48 (1.0)	39 (0.2)		6.93	35 (0.7)	22 (0.1)	
0.22	20 (0.4)	35 (0.2)		9.19	33 (0.7)	18 (0.1)	
0.29	20 (0.4)	35 (0.2)		16.50	25 (0.5)	23 (0.1)	
0.43	16 (0.3)	25 (0.1)					
0.65	16 (0.3)	23 (0.1)					

Try (GI)			195.16	Affect loss (Lasswell)			192.32
0.00	174 (3.5)	1172 (5.8)		0.00	2132 (42.7)	12568 (61.8)	
0.63	3369 (67.5)	16344 (80.3)		0.01	1774 (35.6)	5974 (29.4)	
1.25	628 (12.6)	1797 (8.8)		0.04	627 (12.6)	1183 (5.8)	
2.77	510 (10.2)	761 (3.7)		0.08	283 (5.7)	400 (2.0)	
5.65	207 (4.1)	192 (0.9)		0.17	105 (2.1)	108 (0.5)	
9.51	61 (1.2)	48 (0.2)		0.22	17 (0.3)	35 (0.2)	
17.85	41 (0.8)	38 (0.2)		0.29	20 (0.4)	35 (0.2)	
				0.44	32 (0.6)	49 (0.2)	

Emotion (GI)			188.77	Try (GI)			192
0.00	256 (5.1)	1424 (7.0)		0.00	178 (3.6)	1196 (5.9)	
0.53	3419 (68.5)	16554 (81.3)		0.70	3477 (69.7)	16696 (82.0)	
1.33	761 (15.3)	1776 (8.7)		1.25	533 (10.7)	1450 (7.1)	
2.98	339 (6.8)	432 (2.1)		2.80	507 (10.2)	740 (3.6)	
4.26	89 (1.8)	79 (0.4)		5.67	196 (3.9)	187 (0.9)	
6.08	57 (1.1)	36 (0.2)		9.68	59 (1.2)	45 (0.2)	
10.27	52 (1.0)	29 (0.1)		17.42	40 (0.8)	38 (0.2)	
17.97	17 (0.3)	22 (0.1)					

Power Arenas (Lasswell)			187.07	Well-being psychological (Lasswell)			182.69
0.00	351 (7.0)	2139 (10.5)		0.00	425 (8.5)	2223 (10.9)	
0.20	3166 (63.4)	15348 (75.4)		0.43	3668 (73.5)	16908 (83.1)	
0.26	298 (6.0)	782 (3.8)		0.95	447 (9.0)	824 (4.0)	
0.54	605 (12.1)	1437 (7.1)		1.80	224 (4.5)	232 (1.1)	
1.01	328 (6.6)	438 (2.2)		2.93	124 (2.5)	92 (0.5)	
1.52	132 (2.6)	111 (0.5)		4.72	52 (1.0)	36 (0.2)	
2.34	68 (1.4)	53 (0.3)		7.23	26 (0.5)	21 (0.1)	
3.69	42 (0.8)	44 (0.2)		11.30	24 (0.5)	16 (0.1)	

Anger (EmoLex)			185.5	Respect loss (Lasswell)			181.86
0.00	200 (4.0)	1187 (5.8)		0.00	372 (7.5)	1953 (9.6)	
0.79	3459 (69.3)	16666 (81.9)		0.28	3336 (66.9)	16111 (79.2)	
1.49	533 (10.7)	1441 (7.1)		0.43	333 (6.7)	906 (4.5)	
3.21	432 (8.7)	729 (3.6)		0.92	502 (10.1)	895 (4.4)	
5.96	214 (4.3)	215 (1.1)		1.69	227 (4.5)	303 (1.5)	
10.47	82 (1.6)	68 (0.3)		2.50	106 (2.1)	104 (0.5)	
17.37	49 (1.0)	28 (0.1)		3.66	62 (1.2)	33 (0.2)	
26.70	21 (0.4)	18 (0.1)		5.36	34 (0.7)	32 (0.2)	
				8.32	18 (0.4)	15 (0.1)	

Well-being psychological (Lasswell)			181.03	Anomaly (Lasswell)			175.84
0.00	416 (8.3)	2182 (10.7)		0.00	1172 (23.5)	5513 (27.1)	
0.44	3685 (73.8)	16964 (83.4)		0.01	2728 (54.7)	13041 (64.1)	
0.96	447 (9.0)	811 (4.0)		0.04	660 (13.2)	1379 (6.8)	
1.82	221 (4.4)	235 (1.2)		0.06	150 (3.0)	210 (1.0)	
3.05	123 (2.5)	93 (0.5)		0.09	124 (2.5)	94 (0.5)	
4.83	52 (1.0)	31 (0.2)		0.17	95 (1.9)	72 (0.4)	
7.39	22 (0.4)	21 (0.1)		0.24	19 (0.4)	22 (0.1)	
11.47	24 (0.5)	15 (0.1)		0.32	15 (0.3)	13 (0.1)	
				0.56	27 (0.5)	8 (0.0)	

Anomaly (Lasswell)			179.23				
0.00	1161 (23.3)	5458 (26.8)					
0.01	2732 (54.7)	13096 (64.3)					
0.03	658 (13.2)	1369 (6.7)					
0.06	154 (3.1)	213 (1.0)					
0.08	132 (2.6)	100 (0.5)					
0.16	92 (1.8)	70 (0.3)					
0.30	30 (0.6)	35 (0.2)					
0.42	31 (0.6)	11 (0.1)					

Respect loss (Lasswell)			176.75				
0.00	370 (7.4)	1941 (9.5)					
0.32	3464 (69.4)	16465 (80.9)					
0.43	212 (4.2)	584 (2.9)					
0.92	498 (10.0)	877 (4.3)					
1.68	227 (4.5)	301 (1.5)					
2.43	96 (1.9)	98 (0.5)					
3.53	68 (1.4)	37 (0.2)					
5.05	30 (0.6)	29 (0.1)					
7.31	25 (0.5)	20 (0.1)					

Social (GI)			175.73				
0.00	210 (4.2)	1207 (5.9)					
0.46	3221 (64.5)	15892 (78.1)					
1.16	825 (16.5)	2296 (11.3)					
2.65	487 (9.8)	710 (3.5)					
5.11	162 (3.2)	171 (0.8)					
8.06	53 (1.1)	41 (0.2)					
11.39	32 (0.6)	35 (0.2)					

Politeness (Component scores)			174.95				
0.00	11 (0.2)	106 (0.5)					
9.78	3583 (71.8)	17545 (86.2)					
12.67	257 (5.2)	804 (4.0)					
26.56	625 (12.5)	1291 (6.3)					
49.81	281 (5.6)	399 (2.0)					
85.60	140 (2.8)	121 (0.6)					
142.36	61 (1.2)	54 (0.3)					
231.20	32 (0.6)	32 (0.2)					

a Male (GI) refers to the NLP variable “Male” from the GI database.

**Table 4:** Comparison between AMC and quantile categorizations using bootstrap samples. For each bootstrap sample, we calculated the phi effect size and percentage of times AMC-categorized NLP variables outperformed quantile-categorized NLP variables. The average and standard deviation (SD) of these percentages and phi effect sizes were calculated across 100 iterations.

Number of cases	Number of controls	Average ratio^a^ (SD)	AMC average phi^b^ (SD)	Quantile average phi^c^ (SD)

500	2000	91.9 % (5.7 %)	0.204 (0.018)	0.171 (0.016)
1000	4000	92.1 % (3.8 %)	0.201 (0.014)	0.169 (0.012)

a Average ratio that AMC-categorized variables outperform quantile-categorized variables across 100 iterations of bootstrap.

b Average phi effect size of AMC-categorized NLP variables across 100 iterations of bootstrap.

c Average phi effect size of quantile-categorized NLP variables across 100 iterations of bootstrap.

## References

[1] Curtin SC, Hedegaard H, Ahmad FB, Provisional numbers and rates of suicide by month and demographic characteristics: United States, 2020, NVSS-Vital Statistics Rapid Release (2021). https://archive.hshsl.umaryland.edu/server/api/core/bitstreams/b71e4063-3282-4312-b0f5-3cb3182f85d2/content.

[2] Xu J, Murphy SL, Kochanek KD, Arias E, Deaths: Final data for 2019 (2021). https://stacks.cdc.gov/view/cdc/106058/cdc_106058_DS1.pdf.

[3] Franklin JC, Ribeiro JD, Fox KR, Bentley KH, Kleiman EM, Huang X, , Risk factors for suicidal thoughts and behaviors: a meta-analysis of 50 years of research, Psychol. Bull. 143 (2017) 187. 27841450 10.1037/bul0000084

[4] Ganzini L, Denneson LM, Press N, Bair MJ, Helmer DA, Poat J, , Trust is the basis for effective suicide risk screening and assessment in veterans, J. Gen. Intern. Med. 28 (2013) 1215–1221. 23580131 10.1007/s11606-013-2412-6 PMC3744302

[5] Nock MK, Millner AJ, Ross EL, Kennedy CJ, Al-Suwaidi M, Barak-Corren Y, , Prediction of suicide attempts using clinician assessment, patient self-report, and electronic health records, JAMA Netw. Open 5 (2022) e2144373. 10.1001/jamanetworkopen.2021.44373 PMC8796020 35084483

[6] Kessler RC, Hwang I, Hoffmire CA, McCarthy JF, Petukhova MV, Rosellini AJ, , Developing a practical suicide risk prediction model for targeting high-risk patients in the Veterans Health Administration, Int. J. Methods Psychiatr. Res. 26 (2017) e1575. 28675617 10.1002/mpr.1575 PMC5614864

[7] Levis M, Levy J, Dufort V, Gobbel GT, Watts BV, Shiner B, Leveraging unstructured electronic medical record notes to derive population-specific suicide risk models, Psychiatry Res. 315 (2022) 114703. 10.1016/j.psychres.2022.114703 35841702

[8] Levis M, Levy J, Dent KR, Dufort V, Gobbel GT, Watts BV, , Leveraging natural language processing to improve electronic health record suicide risk prediction for Veterans Health Administration users, J. Clin. Psychiatry 84 (2023) 47557. 10.4088/JCP.22m14568 PMC11157783 37341477

[9] Calvo RA, Milne DN, Hussain MS, Christensen H, Natural language processing in mental health applications using non-clinical texts, Nat. Lang. Eng. 23 (2017) 649–685.

[10] Levis M, Westgate CL, Gui J, Watts BV, Shiner B, Natural language processing of clinical mental health notes may add predictive value to existing suicide risk models, Psychol. Med. 51 (2021) 1382–1391. 32063248 10.1017/S0033291720000173 PMC8920410

[11] Torous J, Larsen ME, Depp C, Cosco TD, Barnett I, Nock MK, , Smartphones, sensors, and machine learning to advance real-time prediction and interventions for suicide prevention: a review of current progress and next steps, Curr. Psychiatry Rep. 20 (2018) 1–6. 29956120 10.1007/s11920-018-0914-y

[12] Martinez VR, Flemotomos N, Ardulov V, Somandepalli K, Goldberg SB, Imel ZE, Atkins DC, Narayanan S, Identifying therapist and client personae for therapeutic alliance estimation, Interspeech (2019) 1901–1905. 36703954 10.21437/interspeech.2019-2829 PMC9875729

[13] Tsui FR, Shi L, Ruiz V, Ryan ND, Biernesser C, Iyengar S, , Natural language processing and machine learning of electronic health records for prediction of first-time suicide attempts, JAMIA Open 4 (2021) ooab011. 10.1093/jamiaopen/ooab011 PMC7966858 33758800

[14] Mitra A, Pradhan R, Melamed RD, Chen K, Hoaglin DC, Tucker KL, , Associations between natural language processing–enriched social determinants of health and suicide death among US veterans, JAMA Netw. Open 6 (2023) e233079. 10.1001/jamanetworkopen.2023.3079 PMC10018322 36920391

[15] Levis M, Dimambro M, Levy J, Shiner B, Using Natural Language Processing to develop risk-tier specific suicide prediction models for Veterans Affairs patients, J. Psychiatr. Res. 179 (2024) 322–329. 39353293 10.1016/j.jpsychires.2024.09.031 PMC11531988

[16] Lu H, Barrett A, Pierce A, Zheng J, Wang Y, Chiang C, , Predicting suicidal and self-injurious events in a correctional setting using AI algorithms on unstructured medical notes and structured data, J. Psychiatr. Res. 160 (2023) 19–27. 36773344 10.1016/j.jpsychires.2023.01.032

[17] Adekkanattu P, Furmanchuk A, Wu Y, Pathak A, Patra BG, Bost S, , Deep learning for identifying personal and family history of suicidal thoughts and behaviors from EHRs, NPJ Digital Medicine 7 (2024) 260. 39341983 10.1038/s41746-024-01266-7 PMC11439010

[18] Young J, Bishop S, Humphrey C, Pavlacic JM, A review of natural language processing in the identification of suicidal behavior, Journal of Affective Disorders Reports 12 (2023) 100507.

[19] Arowosegbe A, Oyelade T, Application of natural language processing (NLP) in detecting and preventing suicide ideation: a systematic review, Int. J. Environ. Res. Publ. Health 20 (2023) 1514. 10.3390/ijerph20021514 PMC9859480 36674270

[20] Li S, Karagas MR, Jackson BP, Passarelli MN, Gui J, Adaptive-mixture-categorization (AMC)-based g-computation and its application to trace element mixtures and bladder cancer risk, Sci. Rep. 12 (2022) 17841, 10.1038/s41598-022-21747-7. 36284198 PMC9596719

[21] DoD VA, Center of excellence for suicide prevention, Joint Department of Veterans Affairs (VA) and Department of Defense (DoD) Mortality Data Repository-National Death Index (NDI), MIRECC website (2020). https://www.mirecc.va.gov/suicideprevention/Data/data_index.asp.

[22] Crossley SA, Kyle K, McNamara DS, Sentiment Analysis and Social Cognition Engine (SEANCE): an automatic tool for sentiment, social cognition, and social-order analysis, Behav. Res. Methods 49 (2017) 803–821. 27193159 10.3758/s13428-016-0743-z

[23] Lasswell HD, Namenwirth JZ, The Lasswell Value Dictionary, New Haven, 1969.

[24] Cambria E, Havasi C, Hussain A, SenticNet 2: A Semantic and Affective Resource for Opinion Mining and Sentiment Analysis, in: FLAIRS, 2012, pp. 202–207.

[25] Mohammad SM, Turney PD, Crowdsourcing a word–emotion association lexicon, Comput. Intell. 29 (2013) 436–465.

[26] Stone PJ, Dunphy DC, Smith MS, The General Inquirer: A Computer Approach to Content Analysis, 1966. 5838381

[27] Hutto C, Gilbert E, Vader: A parsimonious rule-based model for sentiment analysis of social media text, in: Proceedings of the international AAAI conference on web and social media, vol. 8, 2014, pp. 216–225 (1).

[28] Hu M, Bing L, Mining and summarizing customer reviews, in: Proceedings of the tenth ACM SIGKDD international conference on Knowledge discovery and data mining, 2004, pp. 168–177.

[29] Scherer KR, What are emotions? And how can they be measured? Soc. Sci. Inf 44 (2005) 695–729.

[30] Urbanowicz RJ, Moore JH, Learning classifier systems: a complete introduction, review, and roadmap, Journal of Artificial Evolution and Applications 2009 (2009) 1.

[31] Caliński T, Harabasz J, A dendrite method for cluster analysis, Commun. Stat. Theor. Methods 3 (1974) 1–27.

[32] Lomax RG, Statistical Concepts: A Second Course, Lawrence Erlbaum Associates Publishers, 2007.

[33] Kim H-Y, Statistical Notes for Clinical Researchers: Chi-Squared Test and Fisher’s Exact Test, vol. 42, Restorative Dentistry & Endodontics, 2017, p. 152. 10.5395/rde.2017.42.2.152 PMC5426219 28503482

[34] Sullivan GM, Feinn R, Using effect size—or why the P value is not enough, Journal of Graduate Medical Education 4 (2012) 279–282. 23997866 10.4300/JGME-D-12-00156.1 PMC3444174

[35] Mooney CZ, Duval RD, Duvall R, Bootstrapping: A Nonparametric Approach to Statistical Inference, sage, 1993.

[36] Mohammed R, Rawashdeh J, Abdullah M, Machine learning with oversampling and undersampling techniques: overview study and experimental results, in: 2020 11th international conference on information and communication systems (ICICS), IEEE, 2020, pp. 243–248.

[37] Andrade C, Mean difference, standardized mean difference (SMD), and their use in meta-analysis: as simple as it gets, J. Clin. Psychiatry 81 (2020), 10.4088/JCP.20f13681. 32965803

[38] Kessler RC, Bossarte RM, Luedtke A, Zaslavsky AM, Zubizarreta JR, Suicide prediction models: a critical review of recent research with recommendations for the way forward, Mol. Psychiatr. (2019) 1–12. 10.1038/s41380-019-0531-0 PMC7489362 31570777

